# Effects of natural extracts in the treatment of oral ulcers: A systematic review of evidence from experimental studies in animals

**DOI:** 10.4317/jced.58567

**Published:** 2021-10-01

**Authors:** Schilin D. Wen, Eulàlia Sans-Serramitjana, Javiera F. Santander, Mariela R. Sánchez, Paulina Salazar-Aguilar, Andrea B. Zepeda, Susana I. Alvarado, Ignacia B. Miranda

**Affiliations:** 1 Grupo de investigación en Ciencias Aplicadas a la Odontología, Facultad Ciencias de la Salud, Universidad Autónoma de Chile, Chile; 2 Doctoral Program in Morphological Sciences, Universidad de La Frontera, Temuco, Chile; 3 Scientific and Technological Bioresource Nucleus (BIOREN), Universidad de La Frontera, Temuco, Chile; 4 Non-Governmental Organization for Technology and Science Development for Humanity, Chile

## Abstract

**Background:**

To evaluate the clinical and histopathological effects of natural extracts in the treatment of oral ulcers induced in animal experimental models.

**Material and Methods:**

We carried out a search in the Medline, Scopus, WoS and Embase databases from the start of the databases to December 2020, and also made a manual search of the references. The search and selection were carried out by two researchers independently. The inclusion criteria were: experimental studies in animal models, in english, which complied with the study object.

**Results:**

A total of 705 articles were identified. After selection by title, abstract and full text, 19 articles were finally included. Natural extracts of *Jasminum grandiflorum*, *Ficus deltoidea*, curcumin and *Bixina orellana* provoked a significantly greater reduction in the size of the ulcer. Extracts of *Salvatora persica*, *Musa acuminate*, *Ganoderma lucidum* mycelia and *Bixina Orellana*, as well as preparations of Kouyanqing Granule and curcumin, were able to reduce levels of pro-inflammatory cytokines and increase the expression and serum levels of growth factors and anti-inflammatory cytokines. Extracts of *Piper sarmentosus*, *Cannabis sativa* and *Bletilla striata* provoked a reduction in the severity of the histological inflammation. No significant differences were observed compared to controls in the treatments with extracts of *Cannabis sativa*, *Aloe barbadensus* Miller and *Malva sylvestris* in reducing the area of the oral ulcers.

**Conclusions:**

Most of the natural extracts described in this review presented a positive clinical and histological effect on the cicatrisation of oral ulcers induced in animal models.

** Key words:**Recurrent aphthous stomatitis, oral ulcer, plants, herbs, extracts, medicine, treatment.

## Introduction

An oral ulcer is defined as a tissue loss that alters the epithelium and the underlying connective tissues (1). Its aetiology is related to several complex conditions developing in the oral cavity (2). Oral ulcers can be classified into acute or chronic, according to their presentation and progression; acute oral ulcers are characterized by their abrupt onset and short duration, whereas chronic ulcers are associated with slow onset and insidious progression (1). Traumatic ulcers, recurrent aphthous stomatitis, microbial infections and allergic reactions are conditions related to acute mouth ulcers (3-6). In this context, recurrent aphthous stomatitis (RAS) is considered the most common disease of the oral mucosa, with prevalence between 0.9 and 78% (7,8). The prevalence of RAS increases with higher socio-economic status and female gender (9,10). The principal manifestations of this disease are small, round, painful, self-healing ulcers with circumscribed margins, erythematous haloes, and yellow or grey pseudo-membranes (11,12). The management depends on the severity of the lesions, but in all cases the consensus recommendation for treatment is to reduce the pain and duration of ulcers by suppressing the local immune response and preventing secondary infection (13). Topical corticosteroids, topical anaesthetics and analgesics are commonly recommended due to the minimal occurrence of serious adverse effects. In patients with more frequent or severe forms of RAS, systemic immunosuppressive treatment is highly recommended (14); however, long-term exposure to these medications may cause drug resistance, oral flora imbalance, and secondary fungal infection (14,15). In this context, there has been growing interest in the viability of natural extracts as a treatment alternative for RAS due to the lack of adverse effects (15,16). Natural extracts have already proved effective for managing other oral health problems apart from ulcers, such as halitosis and bleeding gums (17), as well as systemic pathologies such as liver, cardiovascular, gastrointestinal and neurological disorders, among others (18-20). Several clinical studies have shown positive effects of natural extracts in reducing the pain and duration of mouth ulcers (21-24). Natural extracts contain several types of secondary metabolites such as flavonoids, polyphenols, and lipophilic, water-soluble polysaccharides; it has been reported that these bioactive ingredients are mainly associated with anti-adherence and anti-inflammatory effects due to their stimulation of the immune response by increasing the production of T-cells and polymorphonuclear neutrophils (PMN), as well as the activation of macrophages and monocytes (25). Although there are descriptions of the use of natural extracts for treating RAS, evidence based on experimental studies in animal models is limited. Animal models are essentially used to understand the process, mechanisms, and aetiology of a disease, as well as to check the safety, efficacy, outcome, and side effects of potential treatments (26,27). The aim of this review was therefore to evaluate the clinical and histopathological effects of natural extracts in the treatment of oral ulcers induced in animal experimental models.

## Material and Methods

-Review protocol

The systematic review protocol was registered in the international prospective register of systematic reviews (PROSPERO) of the National Institute for Health Research database (www.crd.york.ac.uk/prospero), reference code number CRD42020209352. This review was prepared according to the Preferred Reporting Items for Systematic Reviews and Meta-analyses (PRISMA) guideline (28).

-Search strategy

A search was carried out in the MEDLINE, WoS, SCOPUS and EMBASE databases. A manual reference search was also carried out. The database search strategy is detailed in Table 1.

**Table 1:**
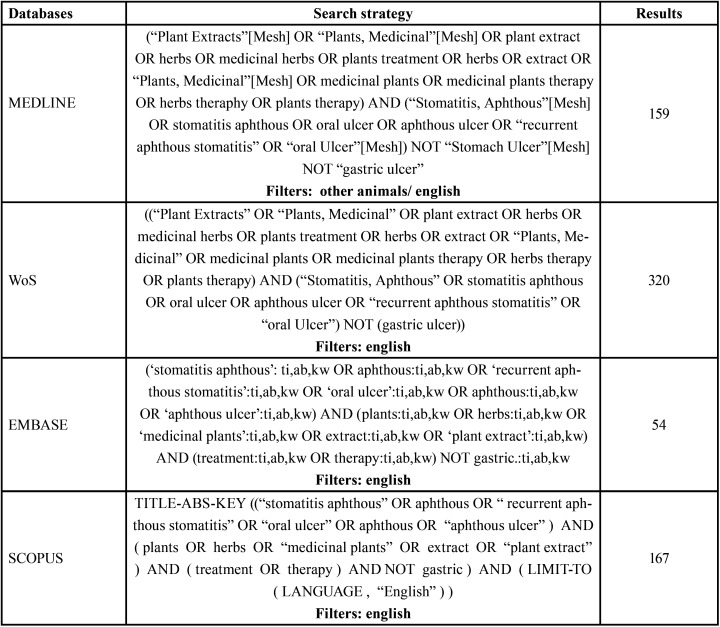
Search strategy.

-Selection criteria

The inclusion criteria were: experimental studies in animal models; in english; available from the beginning of the databases until december 2020; studies that aimed to evaluate the effect of natural extracts in the treatment of oral ulcers.

The exclusion criteria were: reviews, clinical trials, case series, case reports; published in other languages; human or *in vitro* studies; studies to evaluate effect of natural extracts on other pathologies of the oral mucosa.

-Study selection

All the references identified were exported to the Mendeley® reference manager to facilitate the elimination of duplicates. The articles were reviewed by two authors independently (JS, MS); when necessary, a third author (SW) resolved conflicts. Articles were selected first by title and abstract and then by full text, using the Rayyan tool.

-Data extraction

Two data extraction Tables were prepared with the following information: first author and year of publication; total number of subjects and animal species; distribution of the experimental groups; method of induction of oral ulcers in animal model; type of treatment applied (Table 2,  Table 2 cont.); natural extract used as treatment; variables evaluated and results (Table 3,  Table 3 cont.,  Table 3 cont.-1).

**Table 2:**
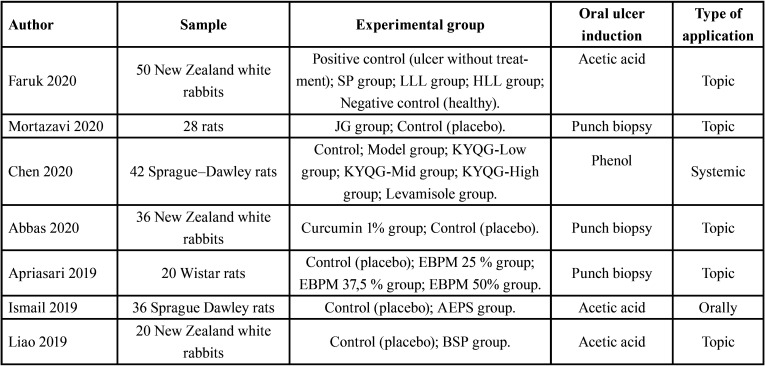
Search strategy.

**Table 2 cont.**
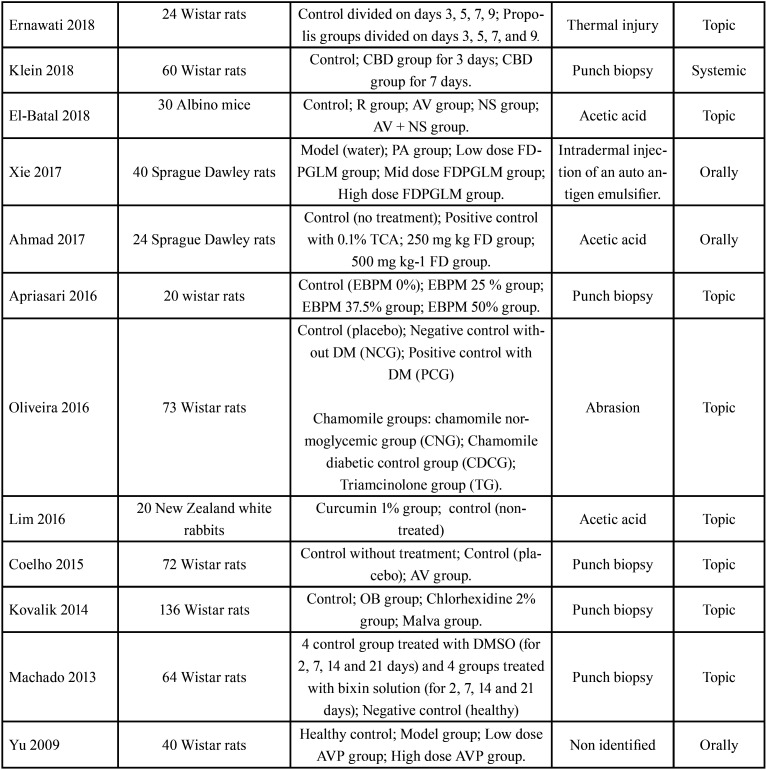
Search strategy.

**Table 3:**
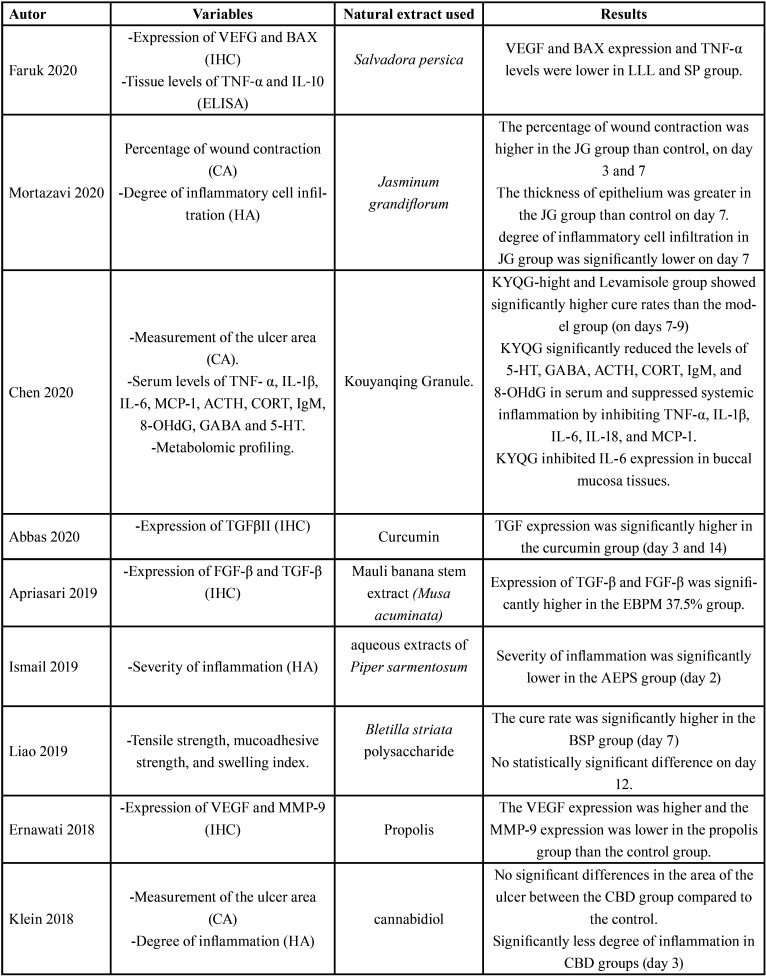
Evaluation of results.

**Table 3 cont.**
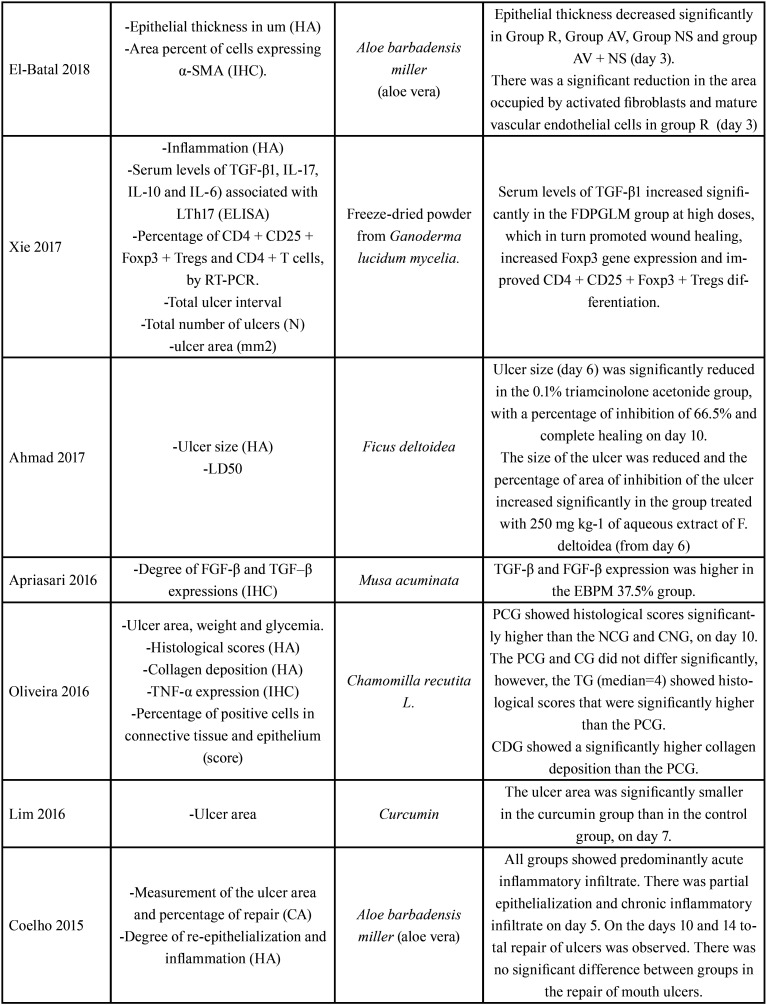
Evaluation of results.

**Table 3 cont.-1:**
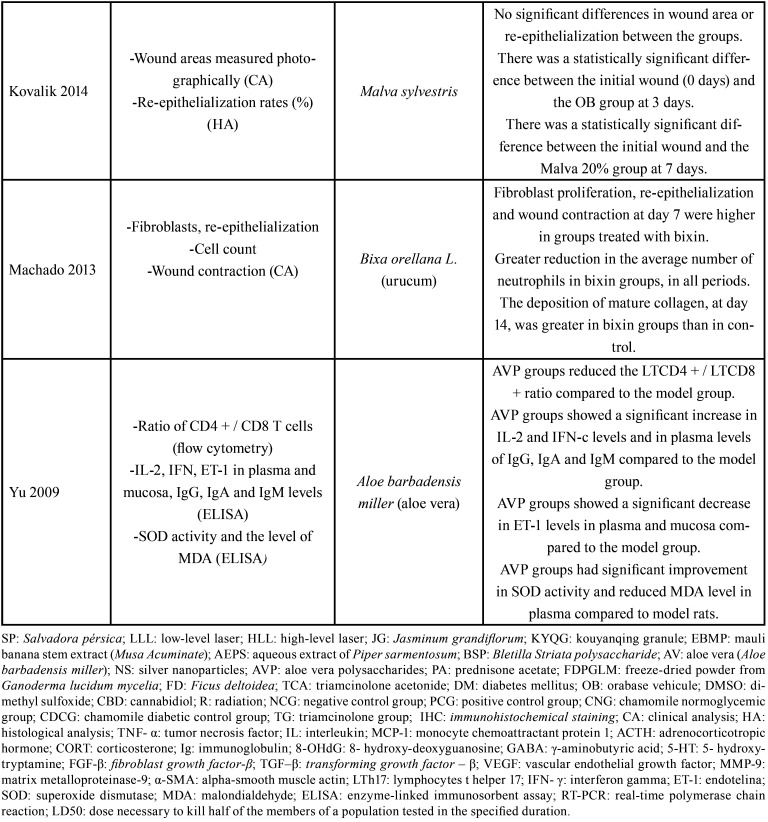
Evaluation of results.

-Risk of bias

Two authors (JS, MS) independently assessed the risk of bias of the articles finally included. A third author resolved conflicts (ES). The Systematic Review Center for Laboratory Animal Experimentation (SYRCLE) guideline (29) was used to assess risk of bias.

## Results

**Figure 1:**
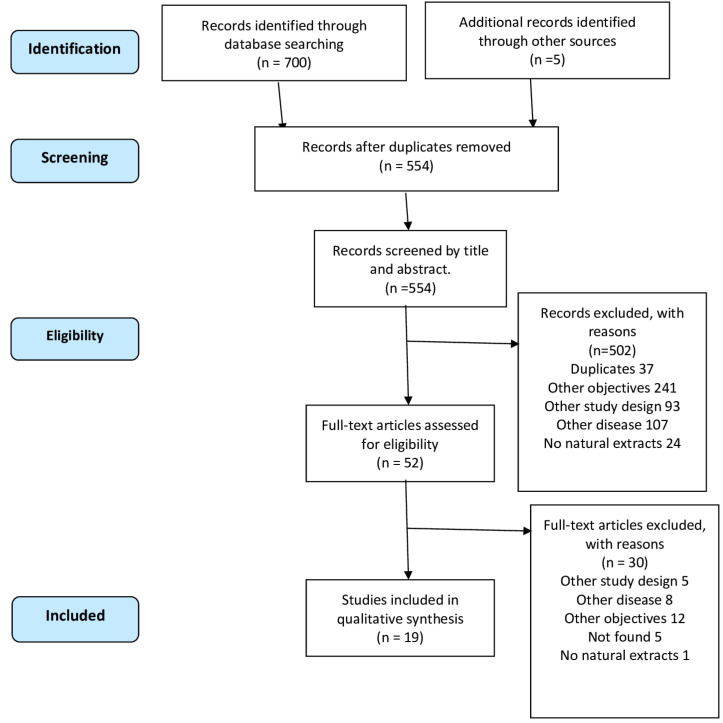
PRISMA Flow Diagram.

A total of 705 articles were obtained; 47 were selected by title and abstract, and 19 articles were finally included by full text (30-48). All the studies had an experimental design in animal models and were published, in english, between 2009 and 2020. The selection process is detailed in Figure 1.

-Extracts used

The extracts used to treat oral ulcers were herbal (34,35,39,42,43,45-48), the fungus *Ganoderma lucidum* (40), propolis (37) and curcumin (33,44). The plants used were of the species *Musa acuminate* (34,42), Aloe barbadensis Miller (45,38,48), Matricaria / Chamomilla recutita (42), *Malva sylvestris* (46), Piper sarmentosum (35), *Jasminum grandiflorum* (31), *Ficus deltoidea* (41), Salvadora persica (30), *Cannabis sativa* (38), *Bletilla striata* (36) and the herbal formula, Kouyanqing Granule (KYQG) (32).

-Ulcer contraction

Seven studies evaluated ulcer area and/or percentage of ulcer contraction. The 57% of the studies observed a significantly smaller ulcer size in the groups treated with extracts of *Jasminum grandiflorum*, *Ficus deltoidea*, Bixa orellana and curcumin compared to controls (31,41,44,47).

-Severity of inflammation

Six studies evaluated the severity and/or degree of inflammation through the presence of inflammatory cells and re-epithelialization. The 66% of the studies observed a significantly lower severity/degree of inflammation in the groups treated with extracts of *Jasminum grandiflorum*, *Pi*per sarmentosum, *Cannabis sativa* and Bixa orellana compared to the controls (31,35,38,47).

-Molecular expression

TGF-β expression was significantly higher in ulcers treated with extracts of curcumin and Musa acuminata (33,34,40) compared to controls. The serum level of TGF-β was also significantly higher in the group treated with *Ganoderma lucidum* extract, compared to the control group (42). TNF-α expression was significantly lower in ulcers treated with extracts of Chamomilla recutita, Salvadora persica and Koyanqing Granule formula, compared to controls (30,32,44). VEGF expression was significantly lower in ulcers treated with Salvadora persica extract and higher in ulcers treated with propolis extract, compared to their respective controls (30,37). The expression of MMP-9 was significantly lower in the ulcers treated with propolis extract, compared to the control group (37). Serum levels of IL-1β, IL-6, IL-18 were significantly reduced in the group treated with Kouyaqing Granule formula compared to the control group (32).

-Risk of bias 

**Figure 2:**
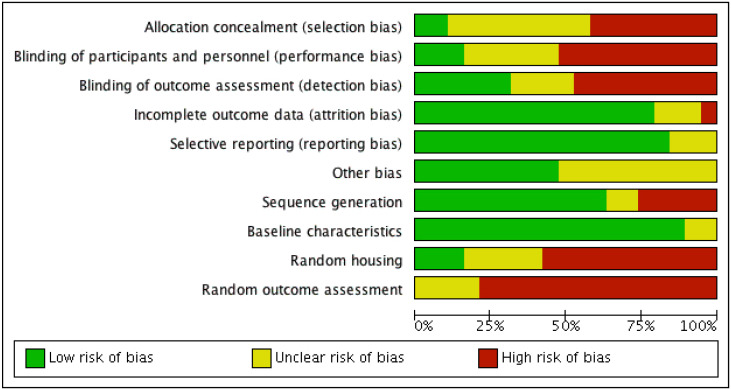
The results of the risk of bias evaluation.

**Figure 3:**
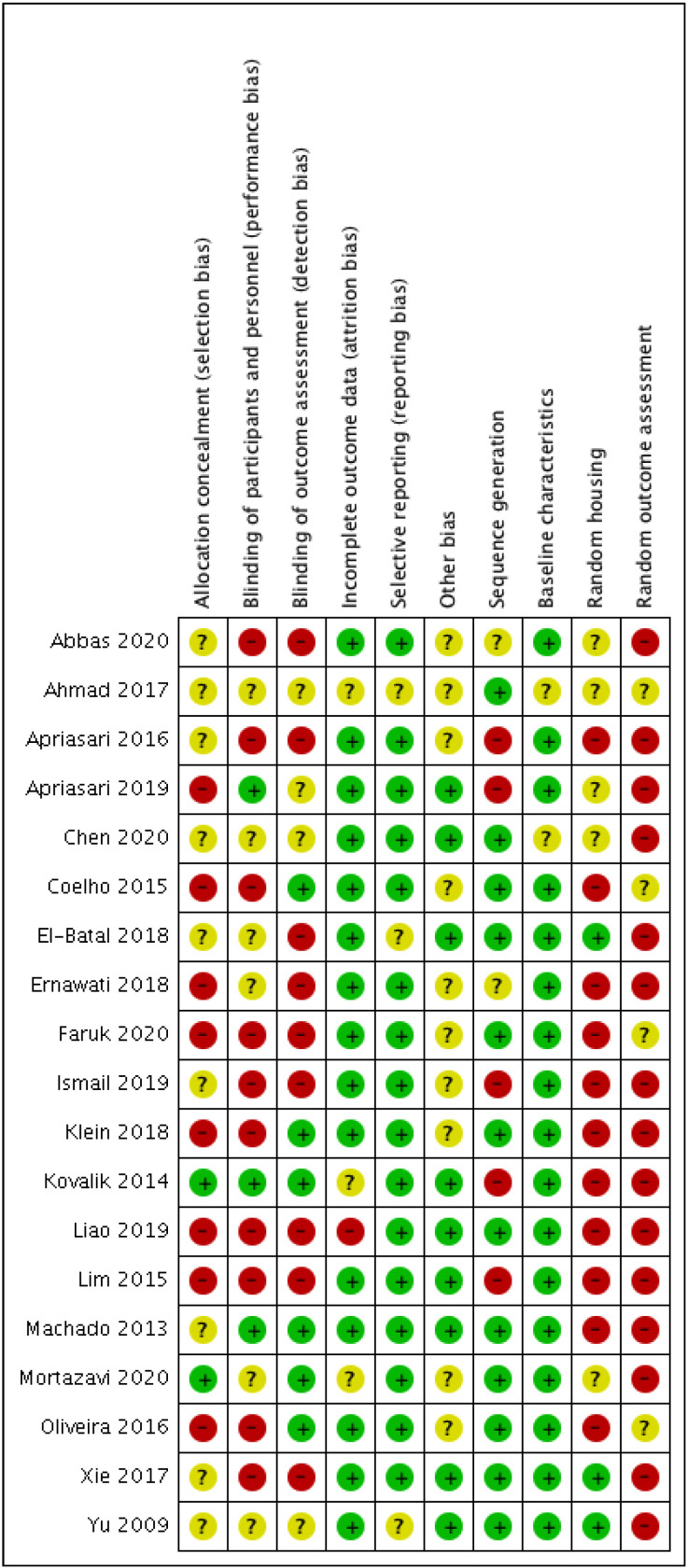
The results of the risk of bias evaluation.

The results of the risk of bias evaluation in the studies selected are shown in Figures 2 and 3.

## Discussion

It has been proposed that natural extracts present multiple health benefits, and can be used effectively for therapeutic ends (49). It is therefore important to consider the scientific evidence available to support their use in the treatment of different diseases. The physiopathology of oral ulcers is complex and comprises vasodilatation, cytokine production, cell death, and tissue repair and remodelling (50). Observations at the molecular level include over-expression of VEGF; over-expression of BAX; marked deposit of collagen fibers in the repair process (30,37); and an increase in both pro-inflammatory and anti-inflammatory cytokines (30,32,37,40,43,48). The 84% of the studies selected reported a positive effect of the treatments on the variables evaluated. The treatments based on curcumin, Musa acuminata and the fungus Ganoderma lucidum showed higher serum levels of TGF-β in comparison with the control groups (ulcers treated with placebo). TGF-β is a cytokine that stimulates the formation of granulation tissue, improves the angiogenic properties of the endothelial progenitor cells to facilitate delivery of blood to the site of the lesion, inhibits matrix metalloproteinase (MMP) and stimulates myofibroblast contraction to allow the wound to close (51).

Extract of the plant Salvadora persica was effective in the repair of induced oral ulcers, provoking a smaller expression of VEGF, TNF-α and IL-10 (30). The propolis-based treatment provoked greater expression of VEGF and smaller expression of MMP-9 in induced oral ulcers (37). This difference in VEGF levels could be explained by the fact that diabetes mellitus (DM) was also induced in these rats, a metabolic pathology in which VEGF levels diminish and MMP-9 levels increase, altering the cicatrisation process (37). Topical propolis gel extract therefore increased the VEGF expression necessary for the repair process, and decreased MMP-9, indicating the presence of angiogenesis; it also decreased collagen degradation, accelerating wound-healing in ulcers in the DM-afflicted rat model.

KYQG is a formulation belonging to traditional Chinese medicine, made up from five different plant species. The rats treated with KYQG were also deprived of sleep for 72 hrs. It was observed that KYQG inhibited the serum levels of IL-1, IL-18, IL-6, monocyte chemoattractant protein 1 (MCP-1) and IL-6 in tissues, and the excessive release of adrenocorticotropic hormone (ACTH) and corticosterone (CORT). It has been shown that lack of sleep can activate the hypothalamus pituitary adrenal (HPA) axis (52). KYQG has also been described as regulating (decreasing) serum levels of γ-aminobutyric acid (GABA) and 5-hydroxytryptamine (5-HT), but it can only diminish the level of 5-HT in the brain. 5-HT is an important pronociceptive mediator which can induce inflammation, hyperalgesia and/or allodynia (43). Extract of *Malva sylvestris*at 20% produced no significant reduction in the area of the oral ulcer, and no positive effect on re-epithelialization of the palatal mucosa in comparison with the control group treated with placebo (46). Extracts of *Jasminum grandiflorum* (29), *Bixina orellana* (45), Musa acuminata stem (32,40), *Bletilla striata* (34), curcumin (42), *Ficus deltoidea*, and *Pi*per sarmentosum were effective in accelerating the repair process; authors observed greater contraction of the ulcer, greater re-epithelialization (31,31,44,47), greater production of factors which accelerate cicatrisation (34), less severe inflammation (37), and better healing rates on days 7 (36,44) and 14 (44). The results with no significant differences obtained by Kovalik *et al*. may be because the animals were kept awake and fed normal food, which could have generated extra mechanical and physical trauma due to mastication. Furthermore, the application of Orabase alone would produce better adherence than the Malva extract gel used (46). Extract of *Bixina orellana* provoked a reduction of neutrophils (cells which secrete elastase, an enzyme that degrades the extracellular matrix (ECM) (47). Treatment with Aloe Vera at 0.5% was not effective in accelerating cicatrisation. This may be explained by the fact that oral mucosa heals more quickly than skin, and perhaps the low concentrations of Aloe Vera are insufficient to stimulate faster recovery in the oral epithelium, which tends to have a much higher basic proliferation index (45). Cannabidiol was able to inhibit chemotaxis and neutrophil proliferation; an anti-inflammatory effect was observed only in the early stage of repair (day 3 after induction of the ulcer), however it did not produce a significant difference in the area of the ulcer. In the initial stage of inflammation, neutrophils release pro-inflammatory cytokines, like TNF-α and IL-β, responsible for increasing vascular permeability, oedema and chemotaxis of the neutrophils; however, their over-expression and production in diabetic patients is related with increased inflammation and delayed cicatrisation of ulcers (38). Freeze-dried powder from G. lucidum mycelia (FDPGLM) in high doses reduced the number and area of oral ulcers induced in rats, increasing significantly the serum levels of TGF- β 1, which in turn promoted cicatrisation of the lesions, increased expression of the Foxp3 gene and improved levels of the lymphocytes CD4+ and CD25+ (40). The majority of the studies analysed in this review report satisfactory results from the use of natural extracts in the treatment of oral ulcers. Their effects are related with reductions in pain and ulcer size, and faster healing. The consensus recommendation for the treatment of oral ulcers is to reduce pain and the duration of the ulcer by suppressing the local immune response and preventing secondary infection (8,13). It is also observed that the principal advantage of using natural extracts is that, unlike their synthetic and chemical counterparts, they do not cause any important secondary effects. For this reason, patients nowadays are tending to change their lifestyles and use natural extracts as a suiTable alternative treatment for oral ulcers.

## Conclusions

Extracts of *Jasminum grandiflorum*, *Bletilla striata*, *Ficus deltoidea*, curcumin, *Bixina orellana*, *Chamomilla recutita* and *Musa acuminate* provoked a positive effect in ulcer contraction, re-epithelialization, and serum levels of molecules that promote repair or accelerate the healing rate. No significant differences from the control groups were observed with the use of treatments based on cannabidiol, Aloe Vera and *Malva sylvestris* in the area of the oral ulcers.
